# Cumulative release characteristics of controlled-release nitrogen and potassium fertilizers and their effects on soil fertility, and cotton growth

**DOI:** 10.1038/srep39030

**Published:** 2016-12-14

**Authors:** Xiuyi Yang, Jibiao Geng, Chengliang Li, Min Zhang, Xiaofei Tian

**Affiliations:** 1 National Engineering Laboratory for Efficient Utilization of Soil and Fertilizer Resources, National Engineering & Technology Research Center for Slow and Controlled Release Fertilizers, College of Resources and Environment, Shandong Agricultural University, Tai’an, Shandong 271018, China; 2 Shandong Provincial Key Laboratory of Water & Soil Conservation and Environmental Protection, Linyi University, Linyi, Shandong 276000, China

## Abstract

To investigate the interacting effects of polymer coated urea (PCU) and polymer coated potassium chloride (PCPC) on cotton growth, an experiment was conducted with containerized plants in 2014 and 2015. There were two kinds of nitrogen fertilizer, PCU and urea, which were combined with PCPC at three application rates (40, 80 and 120 kg ha^−1^). The kinds of nitrogen fertilizer formed the main plot, while individual rates of PCPC were the subplots. The results suggested N and K release patterns for PCU and PCPC in the soil were closely matched to the N and K requirements by cotton. Soil inorganic nitrogen contents significantly increased by using PCU instead of urea, and the same trend was observed with soil available potassium contents, which also had increased rates. Meanwhile, the number of bolls and lint yields of cotton in the PCU treatments were 4.9–35.3% and 2.9–40.7% higher than from urea treatments. Lint yields also increased by 9.1–12.7% with PCPC80 and PCPC120 treatments compared with PCPC40 treatment at the same nitrogen type. Hence, application of PCU combined with 80 kg ha^−1^ of PCPC fertilizer on cotton increased the yields and fertilizer use efficiencies in addition to improving fiber quality and delaying leaf senescence.

Controlled-release fertilizers (CRFs) recently have become popular worldwide because they contain plant nutrients in a form which delays their availability for plant uptake after application, or is available to the plant much significantly longer than a more standard “rapidly available” fertilizer, such as ammonium nitrate, urea, or potassium chloride^1^. Using of CRFs may considerably reduce the energy consumption and time required to grow crops because the nutrients are slowly and gradually released throughout the growing season, hence, only one application is needed. Also consumption of natural gas and waste produced by the fertilizer industry can be reduced because of the more efficient use of nutrients^2^,^3^. However, the use of CRFs is still limited compared with the large amount of more conventional fertilizers applied throughout the world. Relative to non-CRFs, advantages to using CRFs include the ability to obtain a better assessment of expected benefits; improving methods for production of CRFs; optimal design of the fertilizer compositions, inducing synergistic effects; a better understanding of the mechanisms which control nutrient release; and the ability to construct conceptual and mathematical models to predict the release rates and patterns under both laboratory and field conditions. All of these factors may assist growers, technicians, and environmentalists in their decision making^4^,^5^.

Many studies have found that the application of controlled-release urea (CRU) and controlled-release potassium (CRK) greatly improved the yields and fertilizer use efficiencies of crops. Using CRUs have become a new trend to save fertilizer consumption because of the great potential for enhancing fertilizer use efficiencies^6^, reducing environmental pollution^7^,^8^ and saving labor and time^9^. For example, nitrogen release rates of CRUs met the nitrogen requirements and improved apparent nitrogen uptake in wheat in northern China^10^. Similarly, using CRUs increased wheat and maize yields by 12.8–14.3% and 5.5–8.1% compared with normal urea, respectively^11^. Application of CRU also increased the yields and nitrogen use efficiencies in potatoes^12^,^13^. CRUs not only improved yields but also increased the protein content and reduced potential nitrogen losses compared with common urea^14^. The use of CRUs has shown advantages over ammonium nitrate, urea and urea ammonium nitrate, but relative performance varied with rainfall, fertilizer placement and soil texture^15^. Similarly, using CRKs also showed better results compared with conventional potassium in turfgrass^16^. Applications of blended CRK fertilizers may also increase the leaf potassium content in leaves and yield of tobacco^17^. Hence, the use of CRFs should be investigated for possible extensive use in agriculture.

Cotton is an economically important crop globally, and fertilization plays a vital role in improving its seed yield and fiber quality^18^. Nitrogen (N) is a fundamental macronutrient, which is required more consistently and in larger quantities than the other nutrients for cotton production^19^,^20^. Contrary to nitrogen, application of potassium (K) has been neglected in many developing countries, leading to its depletion in many soils, which has prevented increased crop yields^21^. Recently, considerable research has been devoted to studying the effects of CRUs and CRKs on cotton production. For example, application of CRU has been found to enhance the nitrogen supply throughout the cotton growing season thus promoting crop growth^2^. CRU application not only increased nitrogen use efficiencies and crop yields but also reduced the labor costs and risks to the environment^22^. Compared with common urea fertilizers, however, the production costs for CRUs are often somewhat higher although they depend on the specific materials and manufacturing methods^12^. Potassium levels and availability play an important role in cotton production because, similar to nitrogen, a deficiency of potassium can reduce photosynthesis and biomass production, which often results in lower lint yields and poorer fiber qualities^23^. Potassium sulfate (K_2_SO_4_) was usually used as a potassium fertilizer mainly for cotton, although it was less efficient and dearer than potassium chloride (KCl)^24^. Greater efforts should be put into optimizing potassium fertilizer inputs to meet potassium requirements and reduce the costs of cotton production. Hence, a new potassium fertilizer known as polymer coated potassium chloride (PCPC), which was inexpensive, had a high potassium use efficiency, and was easy to apply, and was produced by the National Engineering Laboratory for Efficient Utilization of Soil and Fertilizer Resources in China.

In previous studies on the effects of N-K fertilizers applications on crop growth, positive interactions of N and K have led to lower costs for fertilizers and aided food security^25^,^26^,^27^. A positive interaction of N and K offered the opportunity for considerable savings in the cost of fertilizers and food security for the rapidly expanding human population^23^. Adding larger amounts of potassium fertilizers is a practical way to improve the nitrogen agronomic efficiency, synergistically^28^. Cotton lint yields were both augmented by N and K fertilizer application, and a combination of high plant density, N and K application further improved lint yield in the lower fertility field, while only K application increased lint yield in the higher fertility field^29^. The lint yield, number of bolls, boll weight, lint percentage, fiber qualities, leaf photosynthesis and fertilizer use efficiencies were significantly affected by the types of K, N fertilizers and their interaction^30^. Understanding the mechanisms for N x K interaction is useful in crop production.

Polymer coated urea (PCU), one of CRUs has been widely used, but with a lack of information about the effects of polymer coated potassium chloride (PCPC) and particularly on the PCU × PCPC interaction effects relative to photosynthesis. Hence, we hypothesized that different application rates of PCU and PCPC fertilizers would cause significant differences in fertilizer use efficiencies and cotton yields. The objective of this study was therefore to determine cumulative release characteristics of PCU and PCPC in different media and the influence of PCU and PCPC supplies and their interactions on (i) the contents of soil nitrogen resulting from nitrate (NO_3_^−^-N) and ammonium (NH_4_^+^-N) and the available soil potassium contents; (ii) changes in cotton leaf chlorophyll content using photosynthetic and chlorophyll fluorescence indicators; and (iii) cotton yield, fiber quality and nutrient use efficiency.

## Results

### Cumulative release characteristics of PCU and PCPC in different media

**Figure 1:**
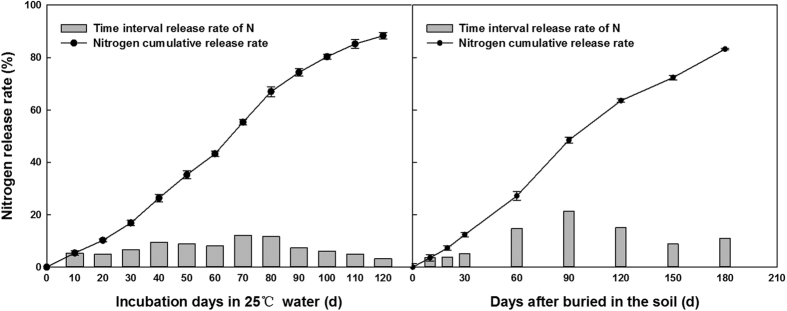
Nitrogen release rate of PCU in water and field condition.

**Figure 2:**
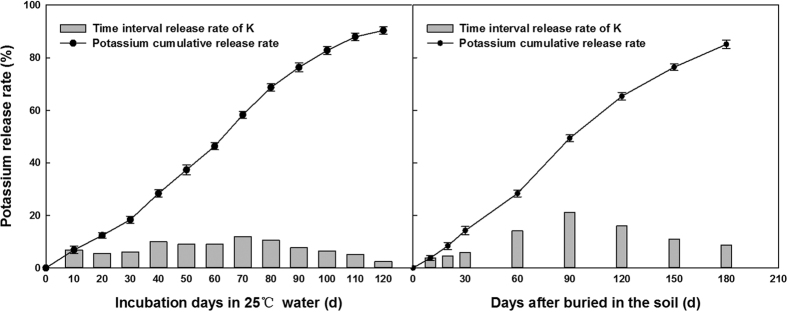
Potassium release rate of PCPC in water and field condition.

The release curve of nitrogen from PCUs under laboratory conditions (in water at 25 °C) revealed a slow stage of release during the first month, followed by more accelerated releasing (40–90 d), and ending with a more decelerate rate of N-release (Fig. 1). Under field conditions, only 12.4% of the nitrogen was released during the first 30 d, then, the release rate accelerated from 60 d to 120 d. By the maturing stage of cotton, 83.2% of the nitrogen had been released. The release rate of potassium from PCPC was also slow during the first 30 d after immersion in 25 °C water, but it then accelerated (40d to 90d), followed by a slower release of potassium (Fig. 2). Under field conditions, only 14.2% of potassium was released during the first 30 d, but it accelerated from 60 d to 120 d. By the harvest stage, 85.1% of the potassium had been released from the PCPC.

### Contents of NO_3_^−^-N, NH_4_^+^-N and available K in soil

**Figure 3:**
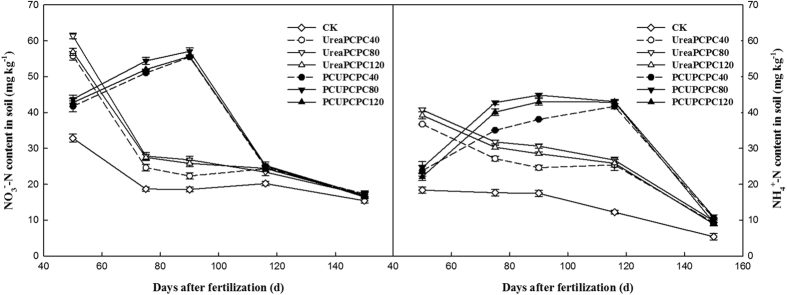
Changes of NO_3_^−^-N and NH_4_^+^-N contents.

**Figure 4:**
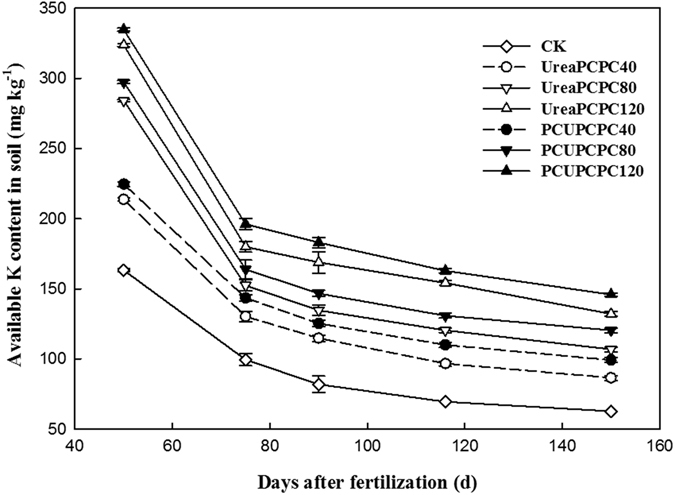
Changes of soil available K content.

The contents of NO_3_^−^-N and NH_4_^+^-N found 0–20 cm deep in the soil were significantly affected by fertilization. The control treatment showed the lowest quantities, which generally decreased in all treatments throughout the cotton growing season (Fig. 3). In the squaring stage, the contents of NO_3_^−^-N and NH_4_^+^-N were each significantly higher in urea than in the PCU treatments, but the concentration decreased rapidly and later became lower than the urea treatment. However a relatively steady nitrogen supply was provided by the PCU treatments during the entire growing season. The contents of NO_3_^−^-N and NH_4_^+^-N were each highest during the full boll stage (57.1 mg kg^−1^ and 44.9 mg kg^−1^, respectively). The potassium fertilizer treatments showed no significant effects on the contents of NO_3_^−^-N or NH_4_^+^-N, which had higher concentrations resulting from the PCPC80 than from the PCPC40 or PCPC120 treatments. The soil available potassium content decreased during the growing season (Fig. 4). For each kind of nitrogen fertilizer, the available potassium concentration increased as the input potassium rate increased. In addition, the levels of soil available K from the PCU treatments were markedly higher than from the urea treatments. Throughout the growing season, the lowest potassium content was observed in the control treatment.

### SPAD values, photosynthetic and chlorophyll fluorescence parameters

**Figure 5:**
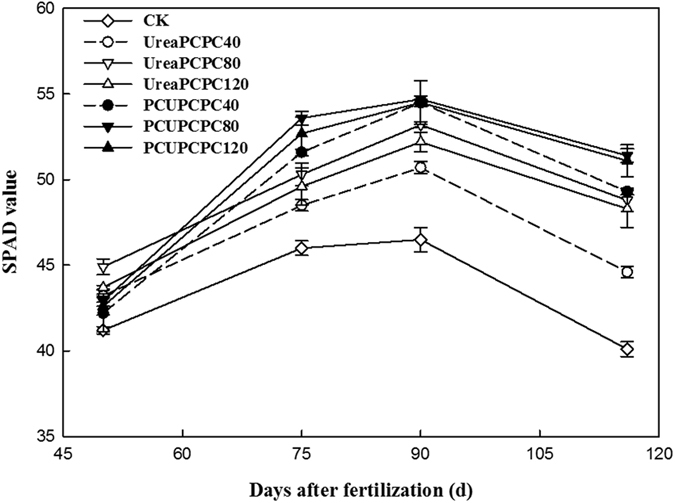
Changes of SPAD value.

**Figure 6:**
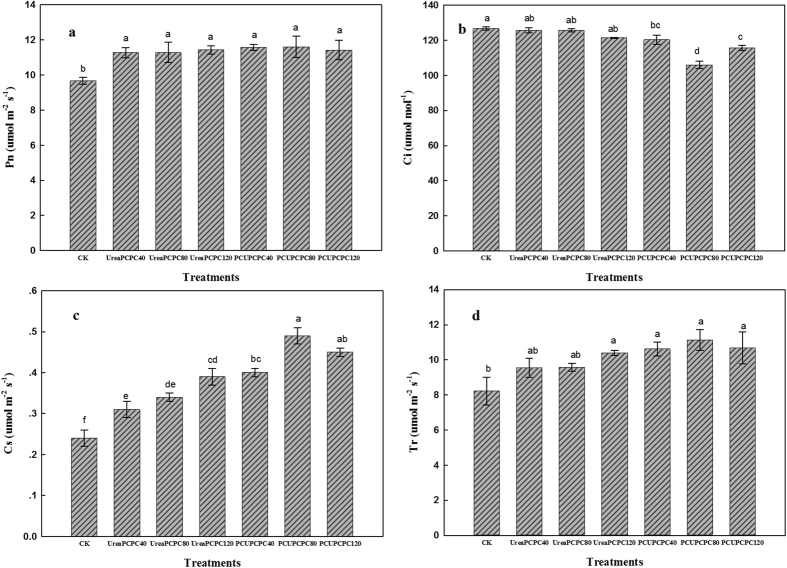
Photosynthesis indicators as affected by N and K fertilization at full boll setting stages.

**Figure 7:**
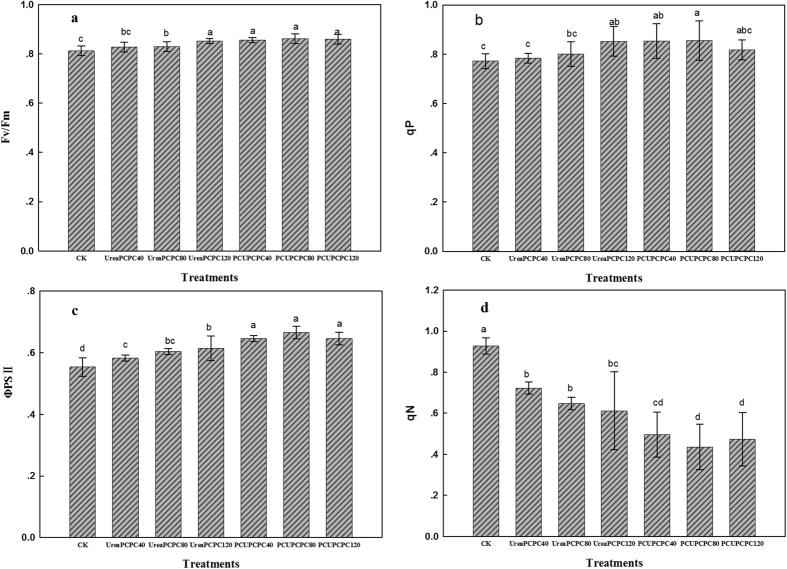
Chlorophyll fluorescence parameters as affected by N and K fertilization at full boll setting stages.

The SPAD values were considerably affected by N and K fertilization with the control treatment yielding the lowest values in each growth stage of cotton (Fig. 5). SPAD values were higher in the squaring stage for treatments of urea than the PCUs, but they decreased rapidly during the season and yielded lower values for urea than the PCU treatments after the first bloom stage. The highest SPAD values occurred in PCU×PCPC80 treatment during the full boll-setting stage. Values for net photosynthesis (Pn), stomatal conductance (Gs) and transpiration (Tr) were each higher, but intercellular CO_2_ concentration (Ci) rate was lower for urea than for the PCU treatments (Fig. 6). Also, the PCU treatments increased the intrinsic PSII efficiency, the light conversion efficiency of PSII in the dark (Fv/Fm), and the coefficient of photochemical quenching (qP) rates, but the coefficient of non-photochemical quenching (qN) was lower than in the urea treatments (Fig. 7). Generally, the PCU×PCPC80 treatment had the most positive effect on SPAD values and on photosynthetic and chlorophyll fluorescence indicators.

### Cotton yield, fiber qualities and nutrient use efficiencies

The number of bolls, yields of seed cotton and lint were all affected by the type of nitrogen fertilizer and the PCPC rates and their interactions (Table 1). All PCU treatments produced significantly higher cotton yields and numbers of bolls compared with the urea treatments. Treatment with PCUs led to significantly higher lint yields (by 15.8–19.1%) compared to urea treatments. Generally, the PCU × PCPC80 treatment produced the highest lint yields for both years, when there were also no significant differences between PCU × PCPC40 and PCU × PCPC120. Meanwhile, the PCU treatments increased the number of bolls by 4.9–35.3% compared with the urea treatments. However, the weights of single bolls showed no significant differences among the fertilization treatments, and the control was the numerically lowest treatment each year. In addition, the lint percentage was not affected by the types of nitrogen fertilizers, rates of potassium fertilizers (except in 2015), or their interactions. Lint percentages persistently remained 43.5 to 44.8% among the different treatments.

Fiber quality appeared to be significantly improved by N and K fertilization compared with the control treatment (Table 2). Based on measurements of fiber length, uniformity, and strength, there were obvious significant differences among the N-fertilized treatments, especially with PCU significantly higher than the urea treatments. Fiber qualities were all affected by N × K interaction results, except for fiber elongation. Fiber lengths and strengths in the PCPC80 and PCPC120 treatments were markedly increased compared with PCPC40.

**Figure 8:**
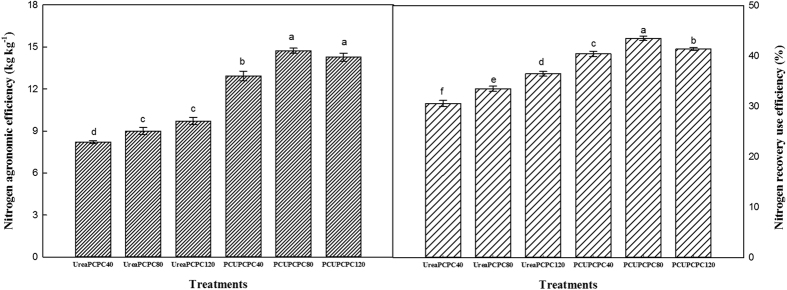
Nitrogen use efficiency as affected by N and K fertilization.

**Figure 9:**
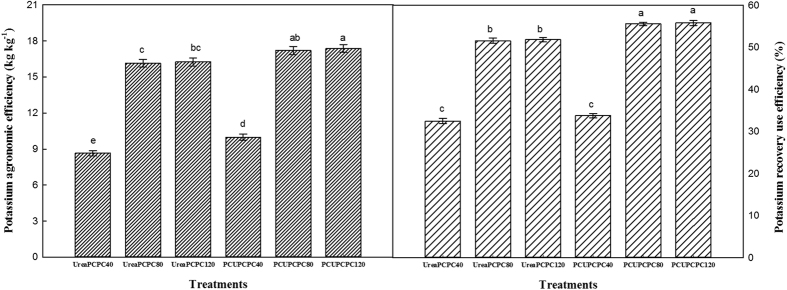
Potassium use efficiency as affected by N and K fertilization.

The nitrogen recovery efficiencies (NREs) in the PCU treatments were all significantly higher than in the urea treatments, and an increasing trend generally occurred with higher rates of urea and PCPC application (Fig. 8). The NREs in PCU treatments were 10.9–42.2% higher than in the urea treatments. Meanwhile, the greatest NRE was produced by PCU × PCPC80 (43.5%), which was significantly higher than in all the other treatments, and was 7.7% and 5.1%, higher than in the PCU × PCPC40 and PCU × PCPC120 treatments, respectively. Similar trends were found for the nitrogen agronomic efficiency (NAE) that the highest NAE also occurred in the treatment with PCU and PCPC80, which reached 14.7 mg kg^−1^. This was the numerically highest treatment NAE and was significantly higher than all other treatments except the PCU and PCPC120. Meanwhile, the potassium recovery efficiency (KRE) and the K agronomic efficiency (KAE) were each significantly higher with increasing PCPC rates, but no significant differences in either variable were observed between PCPC80 and PCPC120 among the treatments with urea or PCU (Fig. 9). The KREs and KAEs from the PCU treatments were increased by 6.6–15.4% and 7.7–38.4%, respectively, compared with the urea treatments provided the same PCPC application rates.

## Discussion

Cotton plants require a continuous supply of nutrients including essential elements for growth, but they absorb them in different quantities and at different speeds during their various developmental stages^31^. The cotton plant was very small and little nutrient was demanded before squaring stage^32^. The successive releases of N and K from PCUs and PCPCs corresponded well with the requirements for N or K during the cotton growth stages^33^,^34^. In the present study, release rates for PCUs and PCPCs were slow before the cotton squaring stage, and then accelerated from the full-bloom to the full-bolling stages, and then decreased during the final stage of cotton maturity. Release rates for the PCUs and PCPCs thus appeared to correspond with the relative rates of cotton N or K uptake during their developmental stages. Meanwhile, the rapid hydrolyses process of urea caused heavy N losses^35^, resulting in lower NO_3_^−^-N and NH_4_^+^-N concentrations compared with the PCU treatments following the squaring stage^36^. The contents of soil NO_3_^−^-N and NH_4_^+^-N significantly increased between the first bloom and the initial boll-opening stages because of the more continuous supplies of nitrogen in the PCU than the urea treatments. Also, the soil available potassium content showed increased concentrations with increasing potassium application rates in any kind of nitrogen treatment. Ammonium-based nitrogen fertilizers can be nitrified after being added to the soil, and when applied at the surface, over half of the urea can be lost through ammonia volatilization^37^,^38^. Direct application of urea also seemed to be resulted in nitrate leaching and potential soil acidification effects. Meanwhile, less-than-ideal nitrogen use resulted in deficiencies adversely affecting cotton growth and yields^39^. Because of its slower release rates, using PCUs reduced soil leaching and denitrification compared with urea. Hence, PCU applications corresponded to 15.8–19.1% higher lint yields than urea. Similarly, an increasing trend with increased potassium fertilization rates with the application of same type of nitrogen fertilization was exhibited. Lint yields and NREs differed significantly with different kinds of N and K rates and their interactions^30^. The main reasons for high KRE and NRE might be more transportation of K and N into cotton bolls by the photosynthesis and less litter during the cotton-growing season. The KRE and KAE of PCU treatments were higher than urea treatments at the same PCPC application rates. Meanwhile, the PCU treatments improved the NRE and NAE compared with urea treatments at different application PCPC rates. Hence, the effects of adding potassium were most relevant when the nitrogen supply was adequate. Also, a positive interaction occurred between the PCU and PCPC treatments based on cotton growth values. Other studies have similarly observed a positive interaction between N and K in increasing crop growth and yields^40^,^41^, and in maize, potassium fertilization has improved NUEs by increasing the rates of nitrogen uptake^42^.

Nutrient supply is a very important factor influencing cotton plant growth^32^. Potassium (K) improves crop quality and protects the plants against abiotic and biotic stresses. Meanwhile, excessive nitrogen dressings have often led to reduced crop quality and higher susceptibility to disease^21^. In the present study, treatment with PCUs corresponded to increased fiber length, uniformity, and strength compared with the urea treatments. This may have resulted from the continuous and adequate supply of nitrogen provided by the PCUs during the key growth stages. However N or K fertilization did not affected cotton micronaire or fiber elongation, which may have been more influenced by genetic regulation than by fertilization; similar effects have been reported in other studies^36^. Fiber qualities improved with increasing K fertilization rates, and a significant N × K interaction effect was found except with fiber elongation. Application of various levels of N or K indirectly affected cotton growth as deficiencies of nitrogen reduced the length, strength, and micronaire of cotton fibers, and potassium stress reduced lint yields and micronaire^25^. Results of the N × K interaction were indicated by higher fiber qualities when cotton was grown with 80 kg ha^−1^ of PCPC. Therefore, application of the PCUs with 80 kg ha^−1^ PCPC will likely produce higher cotton yields and improve fiber quality and nutrient use efficiencies.

Leaf photosynthesis has been closely correlated with leaf senescence, hence, the values for leaf chlorophyll density (SPAD), chlorophyll fluorescence, and photosynthesis of the youngest fully expanded leaves on the main stem have been useful indicators of plant senescence^43^. Leaf senescence has frequently occurred during the rapid filling of cotton bolls and resulted in reduced lint yields and poorer fiber properties^44^. Delayed leaf senescence has corresponded with increased nitrogen fertilization, which likely resulted in improving nutrition and reducing cotton boll loads^45^. Similarly, photosynthesis rates have been positively correlated with application rates of K^24^. In the present study, the application of PCU and PCPC may have delayed the leaf senescence as suggested by the higher indices of photosynthesis (Fig. 7). Also, the slow release of PCU and PCPC may have supplied enough N and K during the entire growth period to increase cotton seed yields and fertilizer use efficiencies. Hence, controlled release potassium fertilizers may effectively promote the growth of cotton, such as in the seedling stage, and improve the leaf physiological characteristics^46^. The PCU treatments delayed leaf senescence and improved cotton plant growth compared with urea. Hence, application of PCU along with 80 kg ha^−1^ of PCPC may delay leaf senescence as suggested by the higher photosynthetic indices we observed.

In summary, lint yields and fiber qualities were significantly affected by the kinds of N and K fertilizers applied, and their interactions. By using PCUs combined with 80 kg ha^−1^ of PCPC fertilizer, maximum lint yields and NREs were achieved, and they were 2.9–40.7% and 19.4–42.2% higher than from the urea treatments, respectively. Because of a continuous supply of nitrogen provided by the PCUs, contents of soil NO_3_^−^-N and NH_4_^+^-N were increased from the first bloom stage to the initial boll-opening stage of cotton. Available soil potassium showed a similar increase that corresponded with higher rates of potassium applied along with any nitrogen treatment. Therefore, fertilization with PCU combined with 80 kg ha^−1^ of PCPC is recommended for maximum yields, fertilizer use efficiencies, and improved quality in cotton cultivation.

## Methods

### Experimental sites and materials

**Figure 10:**
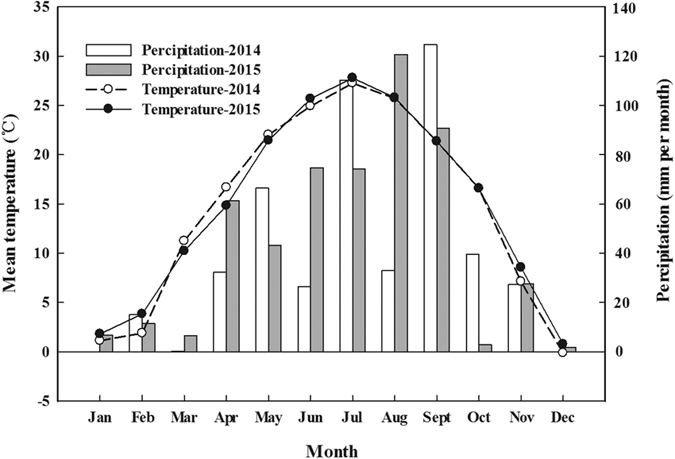
Weather data.

The tests were conducted during two continuous cotton growing seasons (2014–2015) at the New Fertilizer Experiment Station (E 117°13′, N 36°20′), Shandong Agricultural University, in northeast China, with the cotton cultivar ‘Yinshuo 19’. Cotton was grown in pottery pots (height 0.50 m, top diameter 0.50 m, bottom diameter 0.40 m) with a volume of 79 L. To approximate field drainage conditions, there was a hole at the bottom of each pot, and a plastic drain pan was placed beneath the pots to prevent leaching. The soil used in the pots was classified as Coastal Solonchaks, which was a sandy loam with a pH of 8.34, an ECe of 11.2 ds m^−1^, total nitrogen and organic carbon content of 1.15 and 13.6 g kg^−1^, respectively; and levels of NO_3_^−^-N, NH_4_^+^-N, and available P, and K was 14.66, 12.43, 23.12 and 142 mg kg^−1^, respectively. Each pot was filled with 35 kg of air-dried soil initially collected from a cotton field at Dongying city, Shandong Province, China(N 37°49′25″; E 118°29′57″). The climate of the experimental area is temperate and monsoonal, and the weather data is presented in Fig. 10. The conventional fertilizer used was urea (46% N) as N fertilizer and calcium superphosphate (14% P_2_O_5_) as P fertilizer. Controlled release fertilizers included polymer coated urea (PCU, 42% N) and polymer coated potassium chloride (PCPC, 56% K_2_O), which were made by the National Engineering & Technology Research Center for Slow and Controlled Release Fertilizers, China. The PCU and PCPC fertilizers were regularly shaped, rounded particles with smooth surfaces coated with epoxy resin. The coating material accounted for 8.7% and 6.7% of total fertilizer mass. The release longevities for N and K from PCU and PCPC in water (25 °C) were about 3 months.

### Experimental design

A split-plot design with triple replications was used for the study. The main plots were assigned the type of nitrogen fertilizers (90 mg kg^−1^ polymer coated urea: PCU and common urea: Urea), while polymer coating potassium chloride (PCPC) fertilizer rates: 40 (PCPC40), 80 (PCPC80) and 120 (PCPC120) mg kg^−1^ soil, which were assigned to the subplot, and the treatment with no N and K fertilization was as the control. All fertilizers were applied once before sowing seeds. All plots received a basal application of 40 mg kg^−1^ P_2_O_5_ based on local practice. when the cotton plants generally had three true leaves, the plants were thinned to one per pot, and all treatments were measured with the same field management. Otherwise, an automatically device for water monitoring and irrigation was implemented to adjust soil moisture levels based on the changes in plant weight in the test with potted plants^47^.

### Measurement of the N and K content and longevity of PCUs and PCPCs

The N or K release rate in water was determined by the method of “State Standard of the People’s Republic of China for Slow Release Fertilizer”^48^. Here, 10 g of PCU or PCPC was placed in a glass bottle containing 200 ml distilled water with three replicates, and then kept at a constant temperature (25 °C) in an incubator. The released N from PCU was determined by using Kjeldahl method, and the released K from PCPC was measured by a flame photometer, and the solution samples were collected at 10, 20, 30, 40, 50, 60, 70, 80, 90, 100, 110, 120 days or until the accumulative N release rate of PCU and accumulative K release rate of PCPC were more than 80%. For the field conditions, the N and K cumulative release rates were measured by a weight loss method^49^. Specifically, there were 24 mesh bags (the mesh diameter 1 mm, the size of bag is 10 cm length and 8 cm width) each containing 10 g PCU (or PCPC) granules were laced in the ploughed layer of soil before planting cotton in 2015, which was followed by the removal of 3 bags every 10 d during the first month, and then 3 bags every 30 d thereafter. The bags were used for fertilizers, and no soil contained before it loaded in soil. Then, the bags were washed for 60 seconds with deionized water to remove the soil. After that the mesh bags were opened with scissors, PCU (PCPC) particles were removed and washed again with distilled water for 30 seconds. The particles were placed in a vacuum oven at 60 °C for 48 h, and the particles were weighed to determine the rate of N (K) release from PCU (or PCPC)^48^. An electronic balance was used to measure by two correct digits after the dot.

### Soil sampling and measurements

Soil samples were collected at the depth of 0 to 20 cm using a drill (2.0 cm in diameter and 100 cm length) at squaring stage, first bloom stage, full boll setting stage, initial boll-opening stage and full boll-opening stage in the days of 60, 75, 90, 116 and 150 after fertilization in 2015. Soil samples of the same soil depth from three random points were mixed as a composite sample throughout each plot. Then soil samples were collected about 50 g using four sampling techniques, the residual soil samples were filled into soil holes according to the original soil layer. Soil samples were divided into two parts. The concentrations of NO_3_^−^-N and NH_4_^+^-N (extraction with 0.01 M CaCl_2_) were analyzed in extract solution using the AA3-A001-02E Auto-analyzer (Bran-Luebbe, Germany) within 48 h after collection in fresh soil samples. The remainder of the soil samples were air-dried, then sieved (2-mm grid), then the soil-available potassium content was measured by extraction with the CH_3_COONH_4_ method and measured using a flame photometer.

### Chlorophyll content, photosynthetic and chlorophyll fluorescence parameters

The chlorophyll content (SPAD value) was measured with a chlorophyll meter (SPAD-502; Minolta, Tokyo, Japan). Leaf photosynthesis parameters including net photosynthetic (Pn) rate, stomatal conductance (Gs), intercellular CO_2_ concentration (Ci) and transpiration rate (Tr) were determined at the full boll setting stages, using a LI-6400XT portable photosynthesis system (LI-COR, Lincoln, NE, USA) between 09:00 to 11:00 h on a suitable climatic condition. Chlorophyll fluorescence parameters including the primary light energy conversion efficiency of PS II in the dark (Fv/Fm), coefficient of non-photochemical quenching (qN), coefficient of photochemical quenching (qP), intrinsic PS II efficiency (PSII) were measured by using a portable fluorescence system FMS2 (Hansatech instrument, King’s Lynn, Norfolk, UK). The chlorophyll content, photosynthetic and chlorophyll fluorescence parameters were measured at the 3rd fully expanded young leaf, which was on the main stem from terminal per pot.

### Yield and nutrient use efficiency

To measure the cotton yield, the plant per pot was manually harvested by 7 times and weighed after drying in both years. All the bolls were recorded as the boll numbers, which were oven dried to calculate the average boll weight. Lint percentage as well as lint yield was determined after ginning. The fiber samples were sent to Cotton Quality Supervision and Inspection Center at Anyang city, Henan Province in China using the HVI900 analyzer for high volume instrumentation determination of five fiber quality parameters, including micronaire reading, length, strength, length uniformity index, and fiber elongation. All the plant above the root from each pot was sampled, separated them into vegetative organs (stem, leaves and branches), reproductive organs (buds, flowers and bolls), and enveloped them separately. Samples were put into an electric oven for a quick cell killing at 105 °C for 30 min and oven-dried at 85 °C until a constant weight reached before weighed^50^. Plant samples were digested by H_2_SO_4_-H_2_O_2_ mixture and the total N and K concentration of plant were determined by the micro-Kjeldahl procedure and a flame photometer^51^. Aboveground plant N and K uptake were calculated from the sum of the dry matter and both N and K concentration of the different plant parts. The N recovery efficiency (NRE), N agronomic efficiency (NAE), the K recovery efficiency (KRE) and K agronomic efficiency (KAE) were calculated by the following formulas^52^.


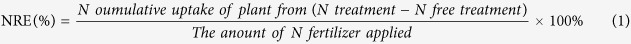



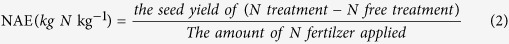



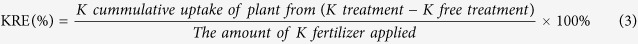



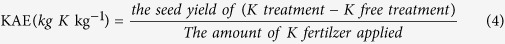


### Data statistical analysis

Microsoft Excel 2007 was employed for data processing, and Sigma Plot software version 10 (MMIV, Systat Software Inc., San Jose, CA, USA) was used to draw the figures. Data was subjected to analysis of variance as a split-plot factorial design with three replications. Two-way analyses of variance (ANOVAs) were performed to determine the effects of PCUs, PCPCs and their interactions on the yield and fiber quality of cotton. One-way analyses of variance were performed to test for significant differences between treatments of NH_4_^+^-N, NO_3_^−^-N, available K, NUE, SPAD values, and photosynthetic and chlorophyll fluorescence parameters. Analyses of variance and mean separation tests (Duncan’s multiple range test, *P* ≤ 5%) were performed using Statistical Analysis System version 9.2 (SAS Institute Cary, NC, 2010), with the function of split-plot design using DPS Data Processing System^53^,^54^.

## Additional Information

**How to cite this article**: Yang, X. *et al*. Cumulative release characteristics of controlled-release nitrogen and potassium fertilizers and their effects on soil fertility, and cotton growth. *Sci. Rep.* **6**, 39030; doi: 10.1038/srep39030 (2016).

**Publisher's note:** Springer Nature remains neutral with regard to jurisdictional claims in published maps and institutional affiliations.

## Figures and Tables

**Table 1:** Main and interaction effects of N and K application on cotton yield and its components.

Treatment	2014					2015				
	Bolls	Boll weight	Seed cotton yield	Lint percentage	Lint yield	Bolls	Boll weight	Seed cotton yield	Lint percentage	Lint yield
	(no. pot^−1^)	(g)	(g pot^−1^)	(%)	(g pot^−1^)	(no. pot^−1^)	(g)	(g pot^−1^)	(%)	(g pot^−1^)
Types of N fertilizers										
Urea	13.2 b	5.66 a	74.73 b	44.49 a	33.26 b	16.3 b	6.55 a	107.40 b	44.39 b	47.69 b
PCU	15.7 a	5.51 a	86.23 a	44.66 a	38.51 a	19.4 a	6.56 a	127.29 a	44.61 a	56.79 a
PCPC fertilizer rates										
PCPC40	13.5 b	5.59 a	75.39 b	44.53 a	33.58 b	17.0 b	6.39 b	108.88 b	44.34 b	48.30 b
PCPC80	15.0 a	5.60 a	83.68 a	44.70 a	37.42 a	18.5 a	6.54 ab	120.97 a	44.62 a	54.00 a
PCPC120	14.8 a	5.56 a	82.38 a	44.49 a	36.65 a	18.2 a	6.74 a	122.20 a	44.54 a	54.43 a
N × K interaction										
Control	11.0 f	5.23 a	57.59 f	43.47 c	25.03 f	11.3 e	5.23 c	59.34 e	43.38 d	25.74 e
Urea × PCPC40	12.0 e	5.69 a	68.26 e	44.36 b	30.28 e	15.0 d	6.28 b	94.10 d	44.21 c	41.60 d
Urea × PCPC80	13.3 d	5.70 a	75.85 d	44.57 ab	33.81 d	16.7 c	6.52 ab	108.54 c	44.45 b	48.25 c
Urea × PCPC120	14.3 c	5.60 a	80.10 c	44.55 ab	35.68 c	17.3 c	6.90 a	119.57 b	44.52 b	53.24 b
PCU × PCPC40	15.0 bc	5.50 a	82.52 bc	44.70 ab	36.89 bc	19.0 b	6.51 ab	123.66 b	44.48 b	55.00 b
PCU × PCPC80	16.7 a	5.35 a	90.88 a	44.79 a	40.71 a	20.3 a	6.46 b	132.43 a	44.80 a	59.76 a
PCU × PCPC120	15.3 b	5.53 a	84.67 b	44.43 ab	37.62 b	19.0 b	6.58 ab	124.83 b	44.56 b	55.63 b
Source of variance										
N	<0.0001	0.2878	<0.0001	0.1593	<0.0001	<0.0001	0.8847	<0.0001	0.0013	<0.0001
K	0.001	0.9742	0.0006	0.2621	0.0004	0.0037	0.0585	0.0002	0.0027	0.0001
N × K	0.0054	0.9034	0.0067	0.2284	0.0037	0.0131	0.1299	0.0005	0.061	0.0004

Note: PCU-polymer coated urea, Urea-common urea fertilizer, PCPC-polymer coated potassium chloride. Means followed by a same lowercase letter in the same column was not significantly different by Duncan’s test in the same year (*P* < 0.05).

**Table 2:** Main and interaction effects of N and K application on fiber qualities.

Treatment	Fiber length (mm)	Fiber uniformity (%)	Micronaire	Fiber elongation (%)	Fiber strength (cN tex^−1^)
Types of N fertilizers					
Urea	27.5 b	83.4 b	5.5 a	6.8 a	28.3 b
PCU	27.7 a	83.9 a	5.5 a	6.8 a	29.3 a
PCPC fertilizer rates					
PCPC40	27.4 b	82.9 b	5.5 b	6.8 a	27.8 b
PCPC80	27.7 a	84.2 a	5.6 a	6.8 a	29.4 a
PCPC120	27.7 a	83.8 b	5.6 a	6.8 a	29.3 a
N × K interaction					
Control	26.7 e	81.3 e	5.2 d	6.7 b	25.2 e
Urea × PCPC40	27.3 d	82.3 d	5.4 c	6.8 a	27.0 d
Urea × PCPC80	27.5 c	83.6 c	5.5 b	6.8 a	28.6 c
Urea × PCPC120	27.8 b	84.2 b	5.6 a	6.8 a	29.4 b
PCU × PCPC40	27.6 c	83.4 c	5.5 b	6.8 a	28.6 c
PCU × PCPC80	27.9 a	84.8 a	5.6 a	6.8 a	30.2 a
PCU × PCPC120	27.6 c	83.5 c	5.5 b	6.8 a	29.2 b
Source of variance					
N	<0.0001	<0.0001	0.0706	0.195	0.0001
K	<0.0001	<0.0001	0.0005	0.2798	<0.0001
N × K	<0.0001	<0.0001	0.0006	0.6243	0.0002

Note: PCU-polymer coated urea, Urea-common urea fertilizer, PCPC-polymer coated potassium chloride. Means followed by a same lowercase letter in the same column was not significantly different by Duncan’s test in the same year (*P* < 0.05).
